# Fully automated rapid quantification of Hepatitis C Virus RNA in human plasma and serum by integrated on-chip RT-qPCR and capillary electrophoresis

**DOI:** 10.1038/s41598-020-64169-z

**Published:** 2020-04-30

**Authors:** Samuel D. H. Chan, Hidenori Toyoda, Jayashree Sanjeeviraman, Aurelie Souppe, Mari Iwamoto, Warren Wu, Daisuke Eto, Toshifumi Tada, Takashi Kumada, Jian-Ping Zhang

**Affiliations:** 1 FUJIFILM Wako Diagnostics U.S.A. Corporation. Mountain View, California, USA; 2 Department of Gastroenterology, Ogaki Municipal Hospital, Ogaki, Japan

**Keywords:** Lab-on-a-chip, Hepatitis C

## Abstract

The quantification of hepatitis C virus (HCV) is essential for the management of chronic hepatitis C therapy. We have developed a fully automated microfluidic RT-qPCR system for rapid quantitative detection of HCV RNA in human EDTA-plasma and serum, and the performance of the method was assessed. The platform for the assay, µTASWako g1 Fully Automated Genetic Analyzer, performs automated sample preparation and RNA extraction, followed by amplification and detection on an integrated RT-qPCR-CE (capillary electrophoresis (CE)) microfluidic chip. The total assay time from sample input to data output is less than 120 minutes. The HCV assay has a linear quantitative range of 15 to 10^7^ IU/mL, with a limit of detection (LOD) of 10.65 IU/mL in EDTA-plasma and 12.43 IU/mL in serum. The assay has a reproducibility of SD ≤ 0.16 log_10_ IU/mL and an accuracy of ≤ 0.22 log_10_ IU/mL difference when compared to the assigned values. The main HCV genotypes 1 to 6 are detected with an accuracy of ± 0.3 log_10_ IU/mL. The assay is specific for HCV RNA and is free of interference from non-HCV pathogens, elevated levels of anti-viral and anti-bacterial drugs, and common endogenous interferents. In the linear quantitative range, the assay is highly correlated with the Roche cobas AmpliPrep/cobas TaqMan HCV Test, version 2.0 (r^2^ = 0.949). As the assay is highly sensitive, accurate and specific, and provides reliable quantification of HCV in plasma and serum, it can potentially be applicable for monitoring the therapy and management of HCV infection.

## Introduction

Hepatitis C virus (HCV) is a single-stranded RNA virus that has been identified as the major etiologic agent responsible for acute or chronic hepatitis^1,2^. A significant number of patients who are chronically infected with HCV will develop liver cirrhosis or hepatocellular carcinoma. Globally, 71 million people have chronic hepatitis C infection and approximately 400,000 people die as a result of infection each year^3^. As HCV does not integrate into the host genome and its propagation relies on continuous replication, the primary goal of antiviral therapy is to achieve sustained virologic response (SVR) and to eliminate the virus from the body of the infected individual. The quantification of HCV viral load before the treatment and monitoring the patient’s response to antiviral therapy are therefore essential for the management of chronic hepatitis C virus infection^4–6^.

Quantitative reverse transcription-polymerase chain reaction (RT-qPCR) has been proven to be the most sensitive method for the quantification of HCV RNA in blood and is used to confirm chronic HCV infection as well as evaluate treatment response to antiviral therapy^5–7^. Two widely used commercial RT-qPCR assays for HCV RNA quantification are the Roche cobas AmpliPrep/cobas TaqMan HCV Test, version 2.0 and the Abbott RealTime HCV Assay^8–10^. Both assays are performed on systems automated for sample preparation, followed by RT-qPCR amplification and detection using fluorescent probes, and they exhibit similar detection sensitivity (LOD ~ 10 IU/mL) and linear dynamic range (~15 to 10^8^ IU/mL). These assays are designed to be high-throughput tests and the high-volume format requires patient samples to be analyzed in bulk, the turnaround time is therefore variable and may require up to a few days from time of blood collection to obtain the result.

We have developed the μTASWako g1 Fully Automated Genetic Analyzer for the quantitative detection of nucleic acids in human clinical samples. The analyzer performs automated sample preparation and nucleic acid extraction, followed by amplification, capillary electrophoresis (CE) and fluorescent detection of the target amplicons. In contrast to existing commercial assays, our system has the ability to analyze a single sample or up to four samples in parallel in a single run, thus facilitating a much more rapid turnover. The system has been used successfully for the detection of Mycobacterium tuberculosis (MTB) and Mycobacterium avium/intracellulare (MAC) in human samples. The corresponding assay kits were recently approved by Japan Ministry of Health, Labor and Welfare and launched in Japan.

In this study, we assessed the analytical and clinical performances of the μTASWako g1 Fully Automated Genetic Analyzer for the quantitative detection of HCV RNA in human EDTA-plasma and serum. The assay requires minimal hands-on time and can be completed within 120 minutes.

## Results

### Real time detection of HCV target amplicon by capillary electrophoresis

**Figure 1:**
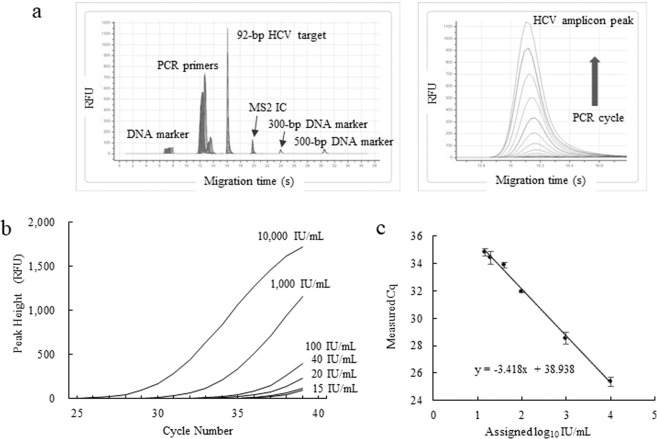
Real time detection of HCV RNA by RT-qPCR-CE. (**a**) Overlay of CE electropherograms for 1000 IU/mL of the 5th WHO International Standard for HCV. (**b**) HCV amplification growth curves. (**c)**, PCR linearity plot. PCR efficiency = 10^(−1/slope)^ − 1 = 96%. RFU stands for Relative Fluorescence Unit.

RT-qPCR assay of the 5th WHO International Standard for HCV spanning the range of 15–10000 IU/mL in EDTA-plasma was performed on the µTASWako g1 Analyzer. Figure 1a shows the overlay of electropherograms of the PCR cycles for 1000 IU/mL of HCV RNA. The graph depicts the growth of the amplicons of HCV RNA target and the non-competitive internal control (IC), MS2 IC as the thermal cycle progressed. Peak alignment was performed using the 300-bp and 500-bp DNA markers. The fluorescent signal was normalized by the peak height of the 300-bp DNA marker. A growth curve for each HCV concentration was generated by plotting the amplicon peak height (Relative Fluorescence Unit) against the PCR cycle number, and the Cq value was determined by using a threshold value of 5. The composite PCR amplification curves and the linearity plot from 15 to 10000 IU/mL of the 5th WHO International Standard for HCV are shown in Fig. 1b,c. The linearity plot, shown with serial dilution of the WHO standard, revealed a linear response over the range of HCV concentration tested. The amplification efficiency was calculated to be 96% (PCR efficiency = 10^(−1/slope)^ − 1).

### Linear dynamic range, traceability to WHO International Standard, and detection limit

**Figure 2:**
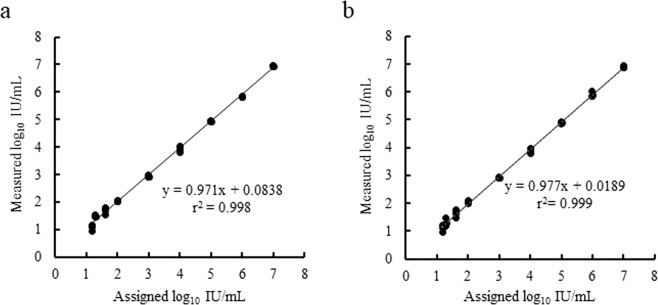
Linear dynamic range of HCV assay. The measured concentration of the HCV Positive Control in (**a**) EDTA-plasma and (**b**) serum spanning the range of 15 to 10^7^ IU/mL was plotted against the assigned value following log_10_ conversion. The measured values (N = 3) for each concentration are within ±0.3 log_10_ IU/mL of the assigned values.

Serial dilutions of the 5th WHO International Standard for HCV and the HCV Positive Control were tested to determine the linear detection range and to provide traceability to the WHO International Standard (Fig. 2). The result showed that the assay had a linear range of 15 to 10^7^ IU/mL. Additionally, all materials tested behaved similarly and demonstrated co-linear dilution performance across the linear range in both the EDTA-plasma and serum matrices. The measured values for each concentration were within the range of ±0.3 log_10_ IU/mL of the assigned values, following log_10_ transformation.

To assess the limit of detection (LOD), seven dilutions of the 5th WHO International Standard for HCV (2.5 to 40 IU/mL) were prepared in HCV negative EDTA-plasma and serum, and the detection rate was determined at each dilution. Probit analysis was performed to determine the LOD (95% detection rate) and the 95% confidence interval. The study demonstrated that the assay was able to detect HCV RNA at a concentration of 10.65 IU/mL with a 95% confidence range of 8.17 to 17.20 IU/mL for 1 mL of the EDTA-plasma specimen, and a concentration of 12.43 IU/mL with a 95% confidence range of 9.22 to 21.76 IU/mL for 1 mL of the serum specimen (Table 1).

**Table 1:** Limit of Detection (LOD) of HCV assay on μTASWako g1 Analyzer.

Input HCV (IU/mL)	Number of valid replicates	Number of valid replicates	Detection rate (%)
**EDTA-plasma**			
40	21	21	100
20	21	21	100
15	21	21	100
10	21	21	100
7.5	21	18	86
5	21	10	48
2.5	21	9	43
0	21	0	0
LOD by Probit at 95% detection rate: 10.65 IU/mL 95% confidence range: 8.17–17.20 IU/mL			
**Serum**			
40	21	21	100
20	21	21	100
15	21	21	100
10	21	19	90
7.5	21	18	86
5	21	11	52
2.5	21	10	48
0	21	0	0
LOD by Probit at 95% detection rate: 12.43 IU/mL 95% confidence range: 9.22–21.76 IU/mL			

### Reproducibility, accuracy, and genotype inclusivity

The reproducibility and accuracy of the assay were assessed by testing serial dilution of the HCV Positive Control from 15 to 10^7^ IU/mL in EDTA-plasma and serum three times. The standard deviation of the measured values in log_10_ IU/mL was ≤ 0.16 (0.03–0.16) across the linear range, indicating good reproducibility of the assay. The accuracy of the assay (Δ log_10_ IU/mL) was within ± 0.22 for both matrices (Table 2).

**Table 2:** Reproducibility and accuracy of HCV assay on µTASWako g1 Analyzer.

HCV input concentration		μTASWako g1 Analyzer measured values							
		HCV log_10_ IU/mL				Δ log_10_ IU/mL			
IU/mL	log_10_ IU/mL	1	2	3	Average	SD	1	2	3
**EDTA-plasma**									
10000000	7.00	6.98	6.97	6.92	6.96	0.03	−0.02	−0.03	−0.0
1000000	6.00	5.84	5.80	5.86	5.83	0.03	−0.16	−0.20	−0.14
100000	5.00	4.92	4.94	4.96	4.94	0.02	−0.08	−0.06	−0.04
10000	4.00	3.82	4.02	4.01	3.95	0.11	−0.18	0.02	0.01
1000	3.00	2.92	2.97	2.92	2.93	0.03	−0.08	−0.03	−0.08
100	2.00	2.06	2.01	2.05	2.04	0.03	0.06	0.01	0.05
40	1.60	1.77	1.53	1.79	1.70	0.14	0.17	−0.07	0.19
20	1.30	1.45	1.49	1.52	1.49	0.04	0.15	0.19	0.22
15	1.18	1.13	1.18	0.95	1.09	0.12	−0.05	0.00	−0.22
**Serum**									
10000000	7.00	6.93	6.87	6.86	6.89	0.04	−0.07	−0.13	−0.14
1000000	6.00	5.84	6.03	5.84	5.90	0.11	−0.16	0.03	−0.16
100000	5.00	4.92	4.88	4.87	4.89	0.03	−0.08	−0.12	−0.13
10000	4.00	3.81	3.99	3.80	3.87	0.11	−0.19	−0.01	−0.20
1000	3.00	2.90	2.89	2.94	2.91	0.03	−0.10	−0.11	−0.06
100	2.00	2.08	1.99	2.06	2.04	0.05	0.08	−0.01	0.06
40	1.60	1.69	1.48	1.77	1.65	0.15	0.09	−0.12	0.17
20	1.30	1.18	1.48	1.23	1.29	0.16	−0.12	0.18	−0.07
15	1.18	1.20	1.18	0.95	1.11	0.14	0.03	0.00	−0.22

**Figure 3:**
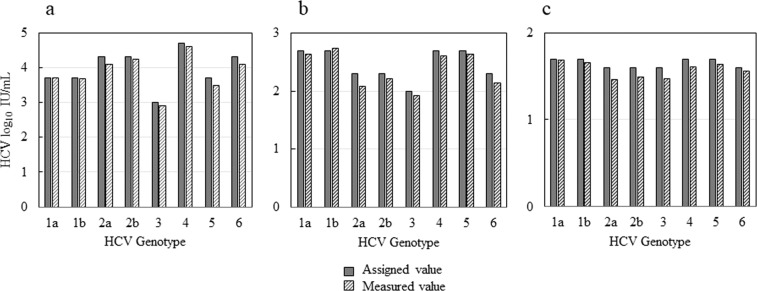
Genotype inclusivity of HCV assay on μTASWako g1 Analyzer. HCV genotype 1a, 1b, 2a, 2b, 3, 4, 5, and 6 in serum were tested at 3 levels (a - high, b - mid, c - low). All genotypes were detected with a mean difference of ≤ 0.3 log_10_ IU/mL between the measured and assigned values (N = 3).

To determine whether the assay covers the main HCV genotypes (genotypes 1–6), three levels of the 5th WHO International Standard for HCV (genotype 1a) and members of the SeraCare HCV RNA Genotype AccuTrak™ Qualification Panel (genotypes 1b, 2a, 2b, 3, 4, 5, and 6) in serum were tested on the system, and the measured log_10_ titers were compared to the respective log_10_ assigned values. All genotypes were detected with a mean difference of ≤ 0.3 log_10_ IU/mL between the measured and assigned values (Fig. 3).

### Confirmation of assay specificity

Potential cross-reactivity was evaluated by testing a panel of pathogens and genomic nucleic acids (Supplementary Table 2) in HCV-negative human serum and human serum containing either the 5th WHO International Standard for HCV or HCV Positive Control. No interference in the performance of the assay was observed in the presence of the added pathogens or nucleic acids for all HCV-negative and positive samples. Elevated levels of triglycerides, bilirubin, serum albumin, hemoglobin, human DNA, the anti-coagulant EDTA (Supplementary Table 3), as well as antidepressants, antiviral and antibacterial drugs (Supplementary Table 4) showed no interference with the assay performance. Finally, the clinical specificity of the assay was confirmed by analyzing 60 HCV-negative EDTA-plasma and 60 HCV-negative serum human specimens as no HCV RNA was detected in any of the samples tested.

### Correlation with Roche cobas AmpliPrep/cobas TaqMan HCV Test, version 2.0 in clinical samples

**Figure 4:**
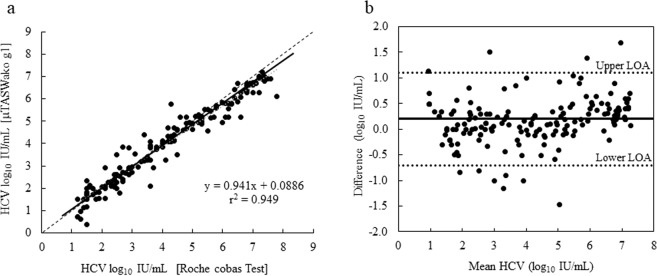
Correlation of HCV assay on μTASWako g1 Analyzer and Roche cobas test. 162 serum samples from HCV-infected patients, previously tested in the Roche cobas test and spreading over the range of 15 to 10^7^ IU/mL, were tested on the μTASWako g1 Analyzer. Deming regression analysis (**a**) shows that the two methods are significantly correlated (r^2^ = 0.949), and the Bland-Altman plot (**b**) indicates that the bias between the two methods are distributed almost evenly in the detection range.

HCV RNA levels in serum specimens of 202 HCV-infected patients were measured by the µTASWako g1 Analyzer and compared to the values determined by the Roche cobas AmpliPrep/cobas TaqMan HCV Test, version 2.0. Deming regression analysis of 162 of the specimens, which spreaded over the linear dynamic range of the HCV assay (15–10^7^ IU/mL, 1.2–7.0 log_10_ IU/mL), showed that the two methods are highly correlated (r^2 ^= 0.949, Fig. 4). A comparison of the remaining 40 specimens, which were either undetectable for HCV RNA or contained less than 15 IU/mL (1.2 log_10_ IU/mL) of HCV RNA measured by the Roche cobas AmpliPrep/cobas TaqMan HCV Test, version 2.0 is shown in Supplementary Table 5. Five of the 21 specimens reported as “Target not detected” by the Roche cobas AmpliPrep/cobas TaqMan HCV Test were tested to be positive (<15 IU/mL) for HCV RNA by the μTASWako g1 Analyzer. Of the 19 specimens tested “Positive and not quantifiable (<15 IU/mL, <1.2 log_10_ IU/mL)” for HCV RNA by the Roche cobas AmpliPrep/cobas TaqMan HCV Test, version 2.0, 12 were tested to contain detectable amount of target by the µTASWako g1 Analyzer.

## Discussion

We evaluated the analytical and clinical performance of the μTASWako g1 Fully Automated Genetic Analyzer, which is built upon a microfluidic platform capable of on-chip RT-qPCR and real time CE separation of amplicons, for the quantitative detection of hepatitis C virus RNA. We showed that the system has excellent specificity, with a lower limit of detection of approximately 12 IU/mL, a broad quantification range of 15 – 10^7^ IU/mL, and precise clinically relevant concentration (±0.30 log_10_ IU/mL) in all HCV genotypes. The system is capable of rapid sample preparation, RNA amplification, and real time quantitative data analysis. The ability to automate the HCV test not only improves the assay performance but also reduces costs by minimizing hands-on time.

This quantitative HCV test utilizes a non-competitive internal control to monitor the entire sample-to-result viral detection process. The internal control MS2 IC is a noninfectious recombinant virus particle and is incorporated into the sample prior to the sample preparation process. As the concentration of the MS2 IC has been optimized and does not interfere with HCV amplification or compromise the detection of low titers samples, the use of this internal control enables the effective monitoring of the whole process of RNA extraction, reverse transcription, PCR amplification and detection. Deviation of the MS2 IC Cq value from the predetermined range will indicate sample loss, a decrease in RNA extraction efficiency, or the presence of substances in the extraction that may be inhibitory to reverse transcription and/or PCR amplification.

In clinical samples obtained from patients with chronic HCV infection, we observed a strong correlation between the HCV RNA levels measured with our method and those from the Roche cobas AmpliPrep/cobas TaqMan HCV Test. This indicates the potential of this method to be used for monitoring of HCV therapy in clinical practice. A key advantage of this rapid method is the total assay time for HCV RNA quantification of less than 120 minutes. Therefore, the information is available to the clinicians with minimal delay after blood collection and sample measurement. According to the American Association for the Study of Liver Diseases (AASLD) guidelines, pre-treatment, on-treatment and post-treatment monitoring of HCV levels in blood are recommended in patients receiving interferon (IFN)-free direct-acting antiviral (DAA) regimens^11^. Timely availability of HCV RNA level will significantly enhance patient care as medical decision-making regarding treatment management can be performed within the same patient visit. In addition, some reports have suggested the potential use of HCV kinetic analyses to individualize the duration of DAA therapy in order to shorten the treatment period^12,13^. Additionally, using this assay to access the very early reduction in serum HCV RNA levels after initiation of antiviral therapy may be useful to monitor treatment effectiveness in a practical way. One of the limitations of this assay system is that the throughput of the instrument is at most 4 samples per run and it takes about two hours per run. However, the results can be utilized for direct medial intervention with minimum reagent and consumable loss since all necessary reagents and consumables per run are supplied on the reagent and consumable cartridge. In the current assay format, a 1 mL patient sample is required for the test and this is another limitation of the assay system, compared with the currently commercially available HCV quantitation systems. With further improvement of the assay detection sensitivity, we believe the input sample volume can be decreased to further enhance the usefulness of the assay in clinical settings.

In conclusion, the data reported here show that the HCV assay on the µTASWako g1 Fully Automated Genetic Analyzer is highly sensitive, accurate and specific for the measurement of HCV levels. Since no interference was observed in the presence of antivirals and antibiotics at concentrations in excess of peak plasma or serum levels, the assay can potentially be used to monitor HCV-infected patients undergoing antiviral therapy. Further clinical evaluation of this HCV test, such as the inclusivity of the assay to a variety of HCV subtypes and uncertainty of the HCV values may support expanding the applications of viral load monitoring and the assurance of the data during the clinical management of chronic hepatitis C virus infection. Moreover, evaluation of the cost effectiveness of the assay is to be conducted to elucidate its clinical significance before the assay is implemented in the clinic.

## Materials and methods

### HCV RT-qPCR primers

The selective amplification of the 92-base HCV RNA target from the patient samples was achieved by the use of a pair of primers specific for the highly conserved 5′- untranslated region (UTR)^14^ of the HCV genome among all the genotypes (forward primer: 5′-TAMRA-ACTGCCTGATAGGGTGCTTG [TAMRA-HCV-267F]; reverse primer: CKTTTGGTTTTTCTTTGAGGT, K = G + T [HCV358R]). The 5′-end of the forward primer carried the fluorescent dye, carboxytetramethylrhodomine (TAMRA), which allowed the HCV amplicon to be labeled for fluorescence detection.

### WHO International Standard for HCV and synthetic HCV Positive Control

The 5th WHO International Standard for HCV (NIBSC code 14/150) was purchased from National Institute for Biological Standards and Control (Hertfordshire, England, UK) and reconstituted in 1.1 mL of nuclease-free water to 100,000 IU/mL (5.00 log_10_ IU/mL). The HCV Positive Control prepared in-house is a non-infectious recombinant MS2 encapsidated construct containing the same 92-base target sequence derived from genotype 1a HCV and is amplifiable by the same set of HCV specific primers for the assay. The concentration of the HCV Positive Control was calibrated with reference to the 5th WHO International Standard for HCV.

### HCV RNA genotype panel

The HCV RNA Genotype AccuTrak™ Qualification Panel (2400-0182) was obtained from SeraCare Life Sciences (Milford, MA, USA). The panel consists of 8 HCV RNA positive plasma samples representing the six genotypes and 4 subtypes (1a, 1b, 2a, 2b, 3, 4, 5, 6) and a negative plasma control. The titer of the samples ranges from 6.82 × 10^4^ to 4.33 × 10^5^ IU/mL.

### MS2 non-competitive internal control (MS2 IC)

MS2 IC is a non-infectious recombinant MS2 phage prepared in-house so that it contains MS2 maturase and coating protein gene and a truncated MS2 replicase gene. It does not contain any HCV RNA sequence and is amplifiable with a pair of primers specific to the replicase gene of MS2. MS2 IC was used as a sample preparation and noncompetitive RT-qPCR internal control and was added to the sample at the beginning of sample preparation to monitor the entire process of the assay, including RNA extraction, reverse transcription, PCR amplification, and detection. The amount of the internal control added into the sample had been optimized such that both the MS2 IC and the HCV RNA targets were independently amplified in the same reaction without interfering with each other. Furthermore, it allowed the MS2 IC to maintain its Cq value with low variability at different target concentrations. As the MS2 IC was taken through the whole sample-to-result process, a delay in its predetermined Cq value would indicate that one or more steps of the process might be compromised and that the assay would need to be repeated.

### Instrumentation

µTASWako g1 Fully Automated Genetic Analyzer is an integrated system containing the components and mechanisms to perform nucleic acid extraction, preparation of the amplification reaction mix, simultaneous target amplification and capillary electrophoresis on a microfluidic chip, and real time detection of amplification products. The heart of the system is a disposable, high-precision molded polycarbonate microfluidic chip containing a 25-µl PCR chamber connected to a network of microfluidic channels^15^. At the end of each PCR cycle, a minute quantity of the fluorescent dye-labeled PCR products is injected from the PCR chamber into the main CE separation channel for analysis, thus generating a PCR amplification curve that reflects the quantitative accumulation of amplicon for each target. The total assay time for HCV RNA detection in human EDTA-plasma or serum on the system is less than 120 minutes. All reagents, consumables and the microfluidic chip are for a single use.

### Automated RNA extraction

MS2 IC was introduced into 1 mL of serum or EDTA-plasma specimen containing the HCV virus. The mixture was treated with proteinase K and chaotropic reagent at elevated temperature to release the nucleic acids. The released and protected RNA were captured on a silica membrane in a column in the presence of isopropanol and washed with wash buffer to remove impurities and potential RT-qPCR inhibitors. The captured RNA was then eluted from the column with elution solution for subsequent on-chip RT-qPCR.

### Automated on-chip RT-qPCR and CE detection

The design of the microfluidic qPCR-CE chip and integrated on-chip PCR and CE for rapid real-time amplicon detection and quantitation have been described previously^15^. For RT-qPCR of HCV RNA, the eluted RNA was mixed with a buffer containing HCV primers, MS2 IC primers, reverse transcriptase, and DNA polymerase. A 25 µL of the reaction mix was added to the PCR chamber of the qPCR/CE microfluidic chip. The primer sequences for the MS2 IC detection are 5′-ATTGCTTACTTAAGGGACGAATTG-3′ and 5′-GCTACGGATGCTGGTTTGT-3′ for forward and reverse, respectively, and the 5′ end of the reverse primer was labeled with TAMRA dye.

Air pressure (138 kPa) was applied to the PCR chamber and reagent wells through a manifold to suppress evaporation and bubble formation during RT-qPCR^15^. Reverse transcription was carried out for 15 minutes at 50 °C, followed by a denaturation and hot start activation step for 4 minutes at 95 °C. Nine short cycles of PCR (6 s at 96 °C, 14 s at 58 °C, and 8 s at 80 °C) were then performed. Afterwards, 29 additional PCR cycles with a longer extension time (38 s at 80 °C) were carried out with one CE injection from the PCR chamber for each subsequent cycle to analyze the accumulated target amplicons.

### Data acquisition and analysis

A PCR amplification curve for each amplicon was generated by plotting the peak height (Relative Fluorescence Unit) versus the cycle number based on the growth of the target peak as the PCR progressed^15^. The quantitative cycle (Cq) values were determined from the amplification curves using a threshold peak height of 5. Cq values were then converted to IU/mL based on a predetermined calibration curve traceable to the 5th WHO International Standard for HCV. The analysis were conducted using a proprietary software developed for the μTASWako g1 instrument.

### Clinical samples from HCV-infected individuals

The assay was validated using 202 clinical serum samples from patients with chronic HCV infection obtained at Ogaki Municipal Hospital, Japan. All patients had tested positive for HCV antibodies and HCV infection had been confirmed by HCV RNA testing. Among the 202 samples, 99 samples were obtained from patients during interferon (IFN)-based anti-HCV therapy and 37 samples were collected from patients during IFN-free anti-HCV therapy with direct-acting antiviral (DAA) agents. The remaining 66 samples were from patients who did not receive any treatment. The HCV RNA levels of the 202 samples, as measured by the Roche cobas AmpliPrep/cobas TaqMan HCV Test, version 2.0, ranged from negative (Target not detected), <15 IU/mL (Positive and not quantifiable) to 7.8 log_10_IU/mL. The HCV RNA negative samples, which totaled 21, were obtained from patients undergoing anti-HCV therapy. The samples were collected from 82 males and 120 females, with a median age of 66 years. The HCV genotype was 1b in 126, 2a in 61, and 2b in 15 patients. Other genotypes were not found as they are very rare in the Japanese population^16^. None of the patients had coinfection with hepatitis B virus or human immunodeficiency virus. Patient backgrounds are shown in Supplementary Table 1. The protocol of this study was approved by the institutional review board of Ogaki Municipal Hospital and carried out in compliance with the Declaration of Helsinki. Written informed consent was obtained from all participants for the use of their clinical and laboratory data, and the stored serum samples. The clinical samples were stored at −80 °C and kept frozen during transport with dry ice prior to the testing.

### HCV-negative EDTA-plasma and serum

60 individual HCV-negative EDTA-plasma and HCV-negative serum specimens were obtained from the American Red Cross (Palo Alto, California, USA). Pooled normal human EDTA-plasma was a product of Golden West Biosolutions (Temecula, California, U.S.A.) and pooled normal human serum was obtained from Golden West Diagnostics (Temecula, California, U.S.A.).

### Statistical analysis

The variability between replicate tests was described using the standard deviation of the log_10_-transformed values. A regression analysis of the log_10_-transformed values was used to analyze the measured and expected values in the study, and to determine the correlation between the HCV assay on the μTASWako g1 Analyzer and the Roche cobas AmpliPrep/cobas TaqMan HCV Test, version 2.0 for the data obtained from the clinical samples.

## Supplementary information

Supplementary Tables.
