# Supplementary material for: Fully automated rapid quantification of Hepatitis C Virus RNA in human plasma and serum by integrated on-chip RT-qPCR and capillary electrophoresis
Source: Sci Rep. 2020 Apr 30;10:7379. doi: 10.1038/s41598-020-64169-z (PMC7193632; doi:10.1038/s41598-020-64169-z)
Supplement: Supplementary file 1 — Supplementary Tables. [file 41598_2020_64169_MOESM1_ESM.pdf]

TITLE

Fully automated rapid quantification of Hepatitis C Virus RNA in human plasma and serum by integrated on-chip RT-qPCR and capillary electrophoresis

AUTHOR LIST:

- Samuel D. H. Chan[1#]
- Hidenori Toyoda[2#]
- Jayashree Sanjeeviraman[1]
- Aurelie Souppe[1]
- Mari Iwamoto[1]
- Warren Wu[1]
- Daisuke Eto[1]
- Toshifumi Tada[2]
- Takashi Kumada[2]
- Jian-Ping Zhang[1]

Supplementary Table 1. Background information of clinical samples from patients with HCV infection for correlation study (n = 202); values shown are median (interquartile range).

Age (years)	66 (59–71)
Gender (Male/Female)	82 (40.6%) / 120 (59.4%)
Alanine aminotransferase (IU/L)	27 (17–40)
Aspartate aminotransferase (IU/lL)	31 (24–46)
γ-glutamyl transpeptidase (IU/lL)	30 (20–47)
Albumin (g/dL)	4.2 (4.0–4.4)
Total bilirubin (mg/dL)	0.7 (0.6–1.0)
HCV genotype (1b/2a /2b)	126 (62.4%) / 61 (30.2%) / 15 (7.4%)
HCV RNA levels - Roche cobas Test (log_{10} IU/mL)	3.4 (1.5–5.5)

Supplementary Table 2. Pathogens tested for cross-reactivity

	Micro-organism	Abbreviation	Description
Virus	*Human immunodeficiency virus 1*	*HIV-1*	HIV-1 High Control, viral particles
Virus	*Hepatitis A virus*	*HAV*	HAV RNA positive sample, 1st International Standard, viral particles
Virus	*Hepatitis B virus*	*HBV*	HBV DNA Positive Control, viral particles
Virus	*Human Adenovirus type 5*	*AdV-5*	Human Adenovirus-5 quantitated viral DNA
Virus	*Human T-lymphotropic virus 2*	*HTLV-2*	HTLV-2 Infected host cell DNA
Virus	*Herpes simplex virus Type 1*	*HSV-1*	Quantitated viral load control - viral particles
Virus	*Herpes simplex virus Type 2*	*HSV-2*	HSV-2 quantitated viral DNA
Virus	*Epstein-Barr virus*	*EBV*	EBV quantitated viral load control - viral particles
Virus	*Human Cytomegalovirus*	*HCMV*	HCMV quantitated viral DNA
Virus	*Human herpes virus type 6*	*HHV-6B*	HHV-6B (Z-29 strain) quantitated viral DNA
Virus	*Influenza A*	*IA (H3N2)*	Genomic RNA from strain A/Hong Kong/8/68
Bacteria	*Propionibacterium acnes*	*P. acnes*	Strain VPI 0389 cell suspension

Bacteria	*Staphylococcus aureus*	*S. aureus*	Genomic DNA from strain Mu50
Yeast	*Candida albicans*	*C. albicans*	Genomic DNA from strain 3147

Supplementary Table 3. Potentially interfering substances (Endogenous Substances)

Triglycerides	15 mg/mL
Bilirubin, conjugated	0.2 mg/mL
Human Serum Albumin	50 mg/mL
Hemoglobin	2.4 mg/mL
Human genomic DNA	1 µg/mL
EDTA	2 - 10 mM

Endogenous substances that are potentially interfering at elevated levels were added to HCV negative and positive (5000 IU/mL) samples at the concentrations shown above. The measurement was done according to the method described in Method section.

Supplementary Table 4. Drug compounds tested for interference

Generic Name	Type of drug	Target/Action	Concentration (1X Cmax)	Concentration (3X Cmax)
Clarithromycin	Antibacterial	Antibacterial	10 µg/mL	30 µg/mL
Ciprofloxacin		Antibacterial	4.56 µg/mL	13.68 µg/mL
Azithromycin		Antibacterial	0.66 µg/mL	1.98 µg/mL
Paroxetine HCl	Antidepressant	Antidepressant	62 ng/mL	186 ng/mL
Fluoxetine HCl		Antidepressant	55 ng/mL	165 ng/mL
Sertraline HCl		Antidepressant	165 ng/mL	495 ng/mL
Entecavir	Nucleoside Analogue	HBV	8.2 ng/mL	24.6 ng/mL
Lamivudine		HBV/HIV	1.5 µg/mL	4.5 µg/mL
Adefovir dipivoxil		HBV	18.4 ng/mL	55.2 ng/mL
Telbivudine		HBV	3.2 µg/mL	9.6 µg/mL
Tenofovir		HBV/HIV	326 ng/mL	978 ng/mL
Interferon alfa-2b	Interferon	HBV/HCV	273 IU/mL	819 IU/mL
Peginterferon alfa-2a		HBV/HCV	28 ng/mL	84 ng/mL
Telaprevir (VX-950)	NS3/4A Protease Inhibitor	HCV	3.51 µg/mL	10.53 µg/mL

Simeprevir (TMC435)		HCV	1.94 µg/mL	5.82 µg/mL
Boceprevir		HCV	1.72 µg/mL	5.16 µg/mL
Sofosbuvir (GS-7977)	Nucleoside and Nucleotide NS5B Polymerase Inhibitor	HCV	603 ng/mL	1809 ng/mL
Ribavirin	Interferon / ribavirin	HCV	3.7 µg/mL	11.1 µg/mL
Peginterferon Alfa-2b		HCV	320 pg/mL	960 pg/mL
Interferon Alfa-2a		HCV	2.58 ng/mL	7.74 ng/mL
Ganciclovir	Compound for treatment of Herpes Virus	Herpes	9 µg/mL	27 µg/mL
Valganciclovir Hydrochloride		Herpes	5.61 µg/mL	16.83 µg/mL
Valaciclovir HCl		Herpes	5.65 µg/mL	16.95 µg/mL
Acyclovir		Herpes	1.61 µg/mL	4.83 µg/mL
Tipranavir, TPV	Protease Inhibitors (Pis)	HIV	57.25 µg/mL	171.75 µg/mL
Indinavir, IDV,		HIV	8.98 µg/mL	26.94 µg/mL
Saquinavir Mesylate, SQV		HIV	2.48 µg/mL	7.44 µg/mL
Lopinavir		HIV	9.6 µg/mL	28.8 µg/mL

Fosamprenavir Calcium salt		HIV	7.24 µg/mL	21.72 µg/mL
Ritonavir, RTV		HIV	11.2 µg/mL	33.6 µg/mL
Darunavir Ethanolate		HIV	6.5 µg/mL	19.5 µg/mL
Atazanavir Sulfate, ATV		HIV	3.15 µg/mL	9.45 µg/mL
Nelfinavir Mesylate, NFV		HIV	4 µg/mL	12 µg/mL
Rilpivirine	Non-nucleoside Reverse Transcriptase Inhibitors (NNRTIs)	HIV	79 ng/mL	237 ng/mL
Etravirine (TMC125)		HIV	1.4 µg/mL	4.2 µg/mL
Delavirdine, DLV		HIV	35 µM	105 µM
Efavirenz, EFV		HIV	4.07 µg/mL	12.21 µg/mL
Nevirapine		HIV	4.5 µg/mL	13.5 µg/mL
Emtricitabine, FTC	Nucleoside Reverse Transcriptase Inhibitor (NRTIs)	HIV	1.8 µg/mL	5.4 µg/mL
Didanosine		HIV	2.32 µg/mL	6.96 µg/mL
Tenofovir Disoproxil Fumarate, TDF		HIV	326 ng/mL	978 ng/mL

Stavudine, D4t		HIV	4.15 µg/mL	12.45 µg/mL
Abacavir Sulfate, ABC		HIV	3 µg/mL	9 µg/mL
Zidovudine		HIV	1.06 µg/mL	3.18 µg/mL
Enfuvirtide, T-20	Fusion Inhibitor	HIV	5 µg/mL	15 µg/mL
Raltegravir (MK-0518)	HIV integrase strand transfer inhibitor	HIV	2.17 µg/mL	6.51 µg/mL
Dolutegravir (GSK1349572)		HIV	4.15 µg/mL	12.45 µg/mL
Maraviroc	Entry Inhibitor – CCR5 co-receptor antagonist	HIV	888 ng/mL	2664 ng/mL
Elvitegravir	Integrase Inhibitor	HIV	1.5 µg/mL	4.5 µg/mL
Cobicistat (GS-9350)	Inhibitor of Cytochrome 450	HIV	1.1 µg/mL	3.3 µg/mL

Drug compounds at concentrations at 1X or 3X of the peak plasma or serum levels were tested in nine pools. The drug compounds were added to HCV negative and positive (5000 IU/mL) samples at the concentrations shown above. The measurement was done according to the procedure.

Supplementary Table 5. Correlation of HCV assay on μTASWako g1 Analyzer and Roche cobas AmpliPrep/cobas TaqMan HCV Test, version 2.0 at low HCV titer

Roche cobas AmpliPrep/cobas TaqMan HCV Test		μTASWako g1 Analyzer	
		Detected	Target not detected
Positive and not quantifiable (< 15 IU/mL)	19	12	7
Target not detected	21	5	16
